# Reward sensitivity and electrodermal responses to actions and outcomes in a go/no-go task

**DOI:** 10.1371/journal.pone.0219147

**Published:** 2019-07-25

**Authors:** Thang M. Le, Wuyi Wang, Simon Zhornitsky, Isha Dhingra, Sheng Zhang, Chiang-Shan R. Li

**Affiliations:** 1 Department of Psychiatry, Yale University School of Medicine, New Haven, Connecticut, United States of America; 2 Department of Neuroscience, Yale University School of Medicine, New Haven, Connecticut, United States of America; 3 Interdepartmental Neuroscience Program, Yale University School of Medicine, New Haven, Connecticut, United States of America; Technion Israel Institute of Technology, ISRAEL

## Abstract

Skin conductance response (SCR) is used in psychophysiological research to measure the reactions of the autonomic nervous system to reward and punishment. While there is consistent evidence that SCR increases to both aversive and appetitive stimuli, it remains unclear whether SCR simply represents a general index of arousal to motivationally significant outcomes or may also differentiate action or inhibition of action that lead to such outcomes. Furthermore, individual differences in trait sensitivity to reward and punishment can influence physiological arousal during approach and avoidance behaviors. Yet, their inter-relationships have not been examined. To address these gaps, we employed a reward go/no-go task with ⅔ go and ⅓ no-go trials and an individually titrated go response window. Correct go and no-go responses were rewarded while incorrect responses were penalized. We examined whether SCR varied with outcome (win vs. loss), action (go vs. no-go), and individual differences in reward sensitivity (SR) and sex. The results showed greater SCRs to loss vs. win, to go vs. no-go success, and to go success in positive correlation with SR. Further, SCR mediated the relationship between SR and go success rate. In sex differences, men exhibited greater SCR which was more predictive of go success rate relative to women. In contrast, SCR was more predictive of no-go success rate in women. Thus, SCR varies according to behavioral contingency, outcome, sex, and reward sensitivity. These findings add to the literature by characterizing the individual and behavioral factors that may influence physiological arousal in response to salient events.

## Introduction

Skin conductance response (SCR) reflects momentary increase in the electrical conductivity of the skin as a result of eccrine sweat gland activity driven by the sympathetic nervous system [1,2]. SCR is modulated by motivationally significant events [3,4] and frequently used as a measure of physiological arousal. For instance, SCRs increase in response to pleasurable stimuli including music [5,6] and happy faces [7] compared to neutral ones. Enhanced SCRs are further associated with reward-related psychological states such as the craving of alcohol [8], cigarette [9,10], and cocaine [11,12] in drug users when exposed to drug vs. non-drug cues. Relative to control stimuli, emotionally negative images [13–16], unpleasant odor [17], physical pain [18], and psychological stress [19] also elicit higher SCRs. Studies directly comparing responses to reward and punishment have found greater autonomic arousal, as measured by SCR, heart rate, and pupil diameter, to monetary losses in comparison to gains [20–22]. The latter findings suggest a negativity bias of physiological response, consistent with the previous proposal that SCR is involved in modulating arousal, attention [23], reasoning [24], and actions [25] during or in anticipation of aversive events. Thus, SCR may not only serve as an index for physiological arousal to valenced outcomes but also reflect different psychological aspects of motivated behaviors that lead to such outcomes.

Actions including motor preparation and movement execution are generally associated with an increase in electrodermal activity [3]. Previous studies of SCRs during tasks that involved motor responses have reported differential patterns of SCR during approach and avoidance behaviors. For instance, SCR was higher for successful go than successful no-go in the go/no-go (GNG) and stop signal tasks [26–29], indicating that it may be more arousing to initiate than to restrain a motor response. Other investigations additionally showed reduced SCRs following avoidance learning and behavior [30,31]. Together with the literature on emotion and reward processing, these findings support the notion that SCR may play an integral role in indexing and potentially linking motivational and motor components of goal-directed actions [32,33]. Yet, no work to our knowledge has directly contrasted the SCR between incentivized response and response inhibition.

Reinforcement Sensitivity Theory suggests individual differences in personality, especially those related to sensitivity to reinforcing events, can influence physiological and behavioral responses to appetitive and aversive stimuli [4,34,35]. In particular, the sensitivity to reward (SR) and punishment (SP), as measured by the Sensitivity to Punishment and Sensitivity to Reward Questionnaire (SPSRQ) [36], have been demonstrated to modulate both physiological responses and tendencies for approach and avoidance behaviors. For instance, higher skin conductance level during exposure to negative images was reported in harm avoidant individuals [37] and predictive of SP and anxiety [38]. Musicians with high approach tendency showed a negative correlation between SP and SCRs evoked during auditory discrimination, as compared to a baseline condition [39]. Sensation seeking, an SR-related trait, was positively correlated with SCR to pleasant images and tendencies for risky behaviors [14]. Those with high vs. low novel seeking trait demonstrated greater SCR to reward than to punishment in the Iowa Gambling Task (IGT) [40]. Further, changes in SCR can precede anticipated outcomes [41,42], indicating the potential involvement of both personality and physiological arousal in shaping behaviors. Indeed, trait anxiety was associated with increased SCR prior to selection from the advantageous deck and overall poorer performance in the IGT [43]. Individuals with psychopathic traits and heightened SR exhibited reduced SCRs to aversive stimuli and increased tendency to engage in disinhibitory behaviors [44,45]. These findings suggest that physiological responses, personality traits, and behaviors may be inter-related. Indeed, earlier studies have used mediation analyses to examine how SCR was associated with borderline personality and conscientiousness to affect behaviors related to interpersonal dysfunction (e.g., aggression, lack of sociability, etc.) [46] and bullying [47], respectively. However, others showed that autonomic responses were related to absolute losses but not to sensitivity to negative outcomes during decision making tasks [20,48]. Thus, the relationship between physiological arousal and trait sensitivity is likely complex and dependent on other variables. It is important to investigate the inter-relationships of trait sensitivity, physiological arousal, and action vs. inhibition of action to better understand how SR and SP influence SCR to motivationally relevant events and contribute to behavioral control.

Sex differences figure prominently in psychophysiological research of motivated behavior [49]. Men are thought to be more motivated than women in the pursuit of reward and women more sensitive to punishment than men [50–52]. Yet, the literature regarding how sex may modulate physiological arousal during motivated behavior has been inconsistent. Studies have found both higher [19,53] and lower [54] SCRs to negative stimuli in women compared to men, as well as lower [55] and higher [49] SCR to positive stimuli in men compared to women. Other studies reported no sex differences in response to either reward or punishment [56–58]. Elliot and Thrash [59] proposed that men are more motivated by approach and women by avoidance goals. A subsequent meta-analysis supported this proposal, showing that men and women are more likely to engage in approach and avoidance behavior, respectively [60]. As investigations examining sex differences in physiological arousal largely focused on passive exposure to emotional imagery without dictating overt actions, it is currently unclear whether behavioral contingencies influence physiological arousal differently in men and women.

Here, we examined the SCR in 67 healthy adults (36 women) during the performance of a GNG task in which successful go and no-go responses were both rewarded and response errors were penalized. Our aim was threefold. Specifically, we sought to (1) characterize how SCR may vary with motivational outcomes and with actions leading to such outcomes, (2) delineate the influence of individual differences in reward and punishment sensitivity and in sex on SCR during incentivized approach and avoidance behavior, and (3) examine the potential inter-relationships between SCR, trait sensitivity, and task performance. As an exploratory aim, we included dollar and nickel trials to determine whether SCR was modulated by reward magnitude. To these ends, we first compared SCR to reward vs. punishment and to action vs. inhibition of action to determine how SCR may serve as an outcome-sensitive measure of arousal in relation to approach and avoidance behaviors. We tested the hypothesis that penalized incorrect responses will be associated with greater SCRs than rewarded correct responses and that rewarded go will exhibit greater SCRs than rewarded no-go. Second, we used the SPSRQ [36] to assess whether SR and SP were predictive of SCR magnitude. We hypothesize that SR and SP will be positively associated with SCR as well as correct response rates of go and no-go trials, respectively. We further conducted mediation analyses to investigate the inter-relationships between trait sensitivity, SCR, and behavioral performance. Finally, we examined sex differences in SCR and related the differences to SP/SR and performance in the GNG task.

## Methods

### Participants, assessment, and behavioral task

Sixty-seven healthy adults (36 women; age = 35 ± 13.9 years, mean ± S.D.) participated in the study. All subjects were screened to be free from major medical, including neurological, illness and Axis I psychiatric disorders. No participants were currently on psychotropic medications and all tested negative for illicit substances on the study day. Subjects provided written informed consent after details of the study were explained, in accordance to guidelines and procedures approved by the Yale Human Investigation Committee.

Participants completed the SPSRQ [36]. The SPSRQ contains 48 yes-no items, with 24 items measuring the scale of behavioral impulsivity/responsiveness to reward and the other 24 measuring the scale of behavioral avoidance in response to potentially adverse consequences. Scores were obtained by totaling the number of yes-answers in each scale, with a higher subscore each indicating higher sensitivity to reward (SR) and sensitivity to punishment (SP).

**Fig 1:**
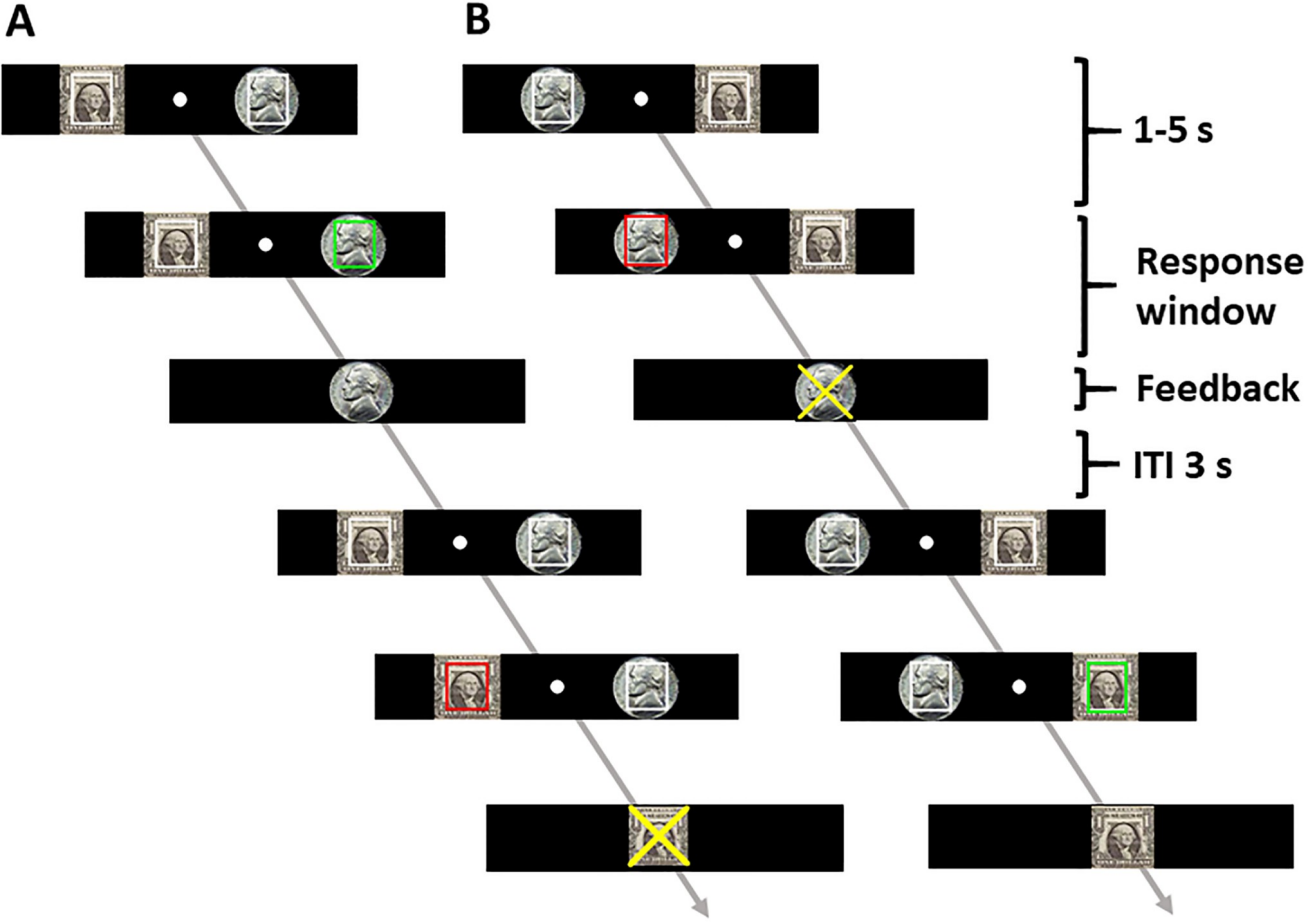
Behavioral task. Participants performed a go/no-go task with the dollar reward on the left (A) and on the right (B), counter-balanced in order across subjects. A successful go trial and failed no-go trial are illustrated in both (A) and (B). Fore-period varied from 1 to 5 s. Response window for go trials was titrated with data obtained from a control session to ensure ~85% success. Inter-trial-interval (ITI) was fixed at 3 s.

Each participant completed four sessions of the go/no-go task (Fig 1). The dollar/nickel rewards were shown to the left/right of the fixation in two sessions and were reversed in location for the other two sessions, with the order counter-balanced across subjects. In each session, go (green square, ~66%) and no-go (red square, ~33%) trials were randomly intermixed in presentation, with an inter-trial-interval of 3 s. Prior to the experiment, each participant received instructions on how to perform the task and completed a control session that did not involve reward. A Gaussian distribution function was fitted on the response time (RT), and 107 data points were generated based on the fitted function. The response window for go success was set as the closest integer greater than 85% of the generated data points for the experiment. The individualized response window was intended to ensure similar, overall monetary wins across subjects.

At the beginning of each trial, two squares outlined in white and overlaid on a dollar/nickel image appeared each to the left and right of fixation. After a randomized interval (fore-period) between 1 and 5 s, one of the squares turned green/red, instructing a go/no-go response. Participants were instructed to press the spatially corresponding left/right button as quickly as possible in response to the go signal, and to withhold the button press to the no-go signal in order to receive the reward. Response window for go trials was titrated with data obtained from control session as detailed above to ensure ~85% success. Feedback was provided at button press or after the response window had elapsed with an image of a dollar or nickel shown to indicate the money won, and an “X” on the dollar or nickel image to indicate the money lost due to incorrect responses. A premature button press prior to the color change would result in an error with the dollar or nickel image overlaid with an X. Participants won an average of $106.6 (± 31.4, mean ± S.D.).

### Skin conductance activity: Acquisition and analysis

We used a Biopac MP150 system to continuously record skin conductance from the palm surfaces of the index and middle fingers of the left hand. The Biopac system used a AcqKnowledge 4.1 software (Biopac Systems, USA) and the Biopac electrodermal activity amplifier module (Galvanic Skin Response 100c) set at a channel sampling rate of 31 Hz and a gain of 5 μSiemens (μS) per volt (resulting in a resolution of 0.0015 μS). Recording of skin conductance was synchronized with the behavioral task. A smoothing function with a moving average of 500 ms was applied in order to eliminate high-frequency noise [61].

Because all trials were longer than 7 s, we used a 7-s window aligned with the stimulus onset to compute the SCR for individual trials, with reference to a 7-s period of an immediately preceding GS trial as baseline. That is, trials were included in the analyses if they were immediately preceded by a GS trial, which represented the most frequent trial type. The SCR of each trial was computed by subtracting the mean over the 7-s baseline from its peak skin conductance value. Thus, every trial was individually baseline-corrected, ensuring SCRs were not affected by potential non-task-related fluctuations in skin conductance over multiple trials and task sessions [62,63]. Data from 7 subjects were not available for the NGE as they did not commit any no-go errors. Across subjects, 16.0 ± 3.0 (mean ± SD) trials were available for GS, 16.0 ± 2.0 for NGS, 15.3 ± 1.4 for GE, and 8.3 ± 3.9 for NGE trials.

### Mediation analysis

We performed mediation analyses to examine the inter-relationships of trait sensitivity, SCR, and go success rate (see Results), using a single-mediator model [64]. The methods were detailed in our previous work [65,66]. Briefly, in a mediation analysis, the relation between the independent variable X and dependent variable Y; that is, X → Y is tested to determine whether it is significantly mediated by a variable M. The mediation test is performed using the following three regression equations:

$$Y=i_{1}+cX+e_{1}$$

$$Y=i_{2}+c^{\prime }X+bM+e_{2}$$

$$M=i_{3}+aX+e_{3}$$

where *a* represents X → M, *b* represents M → Y (controlling for X), *c'* represents X → Y (controlling for M), and *c* represents X → Y. *a*, *b*, c, and *c'* are referred to as “path coefficients” or simply “paths”. Variable M is said to be a mediator of connection X → Y, if (*c*–*c'*), which is mathematically equivalent to the product of the paths *a* × *b*, is significantly different from zero [64]. If (*c*–*c'*) is different from zero and the paths *a* and *b* are significant, then X → Y is mediated by M. In addition, if path *c'* is not significant, there is no direct connection from X to Y, indicating X → Y is completely mediated by M. Note that path *b* represents M → Y, controlling for X, and should not be confused with the correlation coefficient between Y and M. The analysis was performed with package Lavaan [67] in R (https://www.r-project.org).

Three models were considered to examine the inter-relationships of SCR to GS trials, task performance (GS rate) and SR (see Results). In Model 1, SCR was the independent variable (X), task performance was the dependent variable (Y), and SR was the mediator (M). In Model 2, SR, task performance, and SCR served as X, Y, and M, respectively. In Model 3, SR, SCR, and task performance served as X, Y, and M, respectively. The other 3 potential models were not considered due to the lack of conceptual import: GS rate as a performance variable was unlikely an independent variable and SR was unlikely a dependent variable. Task performance and SCR were the correct response rate and SCR of the GS reward trials. To test the significance of the mediation effect, we used the bootstrapping method [68] as it is generally considered advantageous to the Sobel test [64].

The single-mediator model [64] makes three assumptions: 1) residuals in Equations 2 and 3 are independent, 2) M and the residual in Equation 2 are independent, and 3) there is not an X×M interaction in Equation 3. However, these assumptions are untestable in most situations [64] as is the case in the current study, and are generally relaxed in implementation [69,70]. To ensure the validity of the models, we relied on previous work establishing the relationships between the variables (i.e., trait sensitivity, SCR, and motivated approach behavior).

## Results

### Behavioral performance

**Fig 2:**
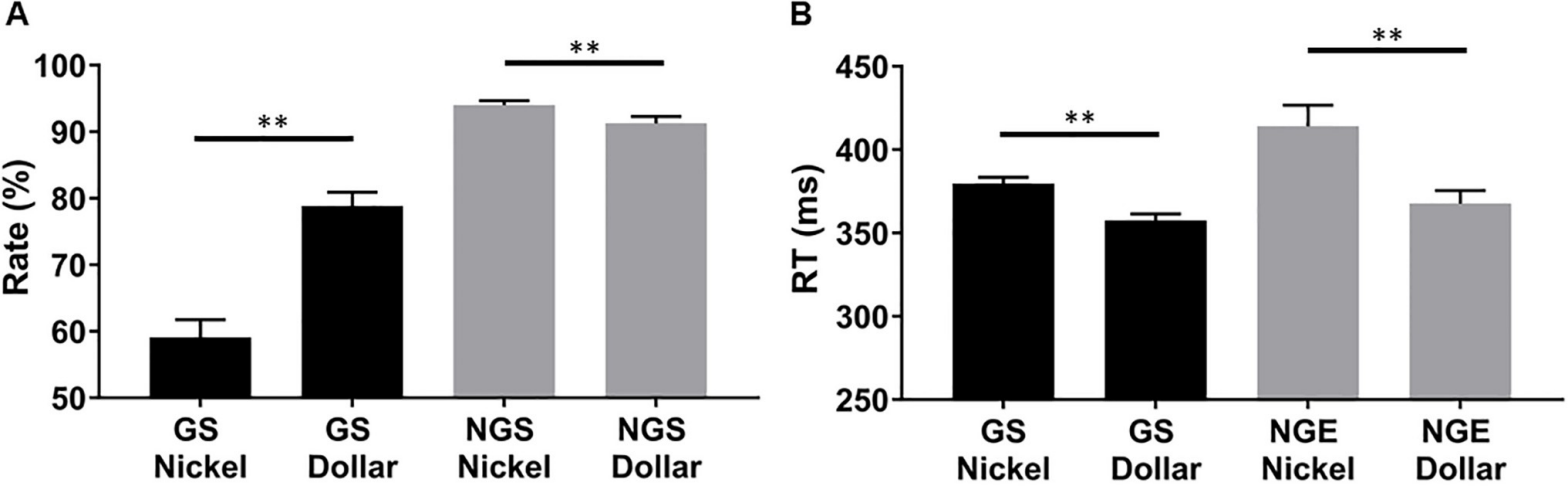
Behavioral results. (mean ± s.e.) of the rate (**A**) and response time (**B**) across trial types. GS: go success; NGS: no-go success; NGE: no-go error. RT: response time. ** *p* ≤ .001.

Fig 2A shows the correct response rate and RT across conditions. For correct response rate, a two-way (GS vs. NGS × dollar vs. nickel) ANOVA showed a significant main effect of response (*F*(1,66) = 171.43, *p* < .001), reward value (*F*(1,66) = 22.05, *p* < .001), and a significant response × reward value interaction (*F*(1,66) = 38.70, *p* < .001). Post hoc analyses revealed GS dollar rate to be higher than GS nickel rate (*p* < .001) but lower than NGS dollar rate (*p* < .001). NGS nickel rate was significantly higher than NGS dollar (*p* = .001) and GS nickel (*p* < .001) rate.

For RT, 22 subjects did not commit any go or no-go errors in either the dollar or nickel trials and thus were not included in the analysis. A two-way (GS vs. NGE × dollar vs. nickel) ANOVA showed a significant main effect of response (*F*(1,44) = 8.91 *p* = .003) and reward value (*F*(1,44) = 19.85, *p* < .001) but not response × reward value interaction (*F*(1,44) = 2.68, *p* = .10). RT was significantly faster in the GS dollar as compared to the GS nickel trials (*p* < .001) (Fig 2B). RT was significantly faster for NGE dollar than NGE nickel trials (*p* < .001). These results suggested participants’ attention was biased toward directional response to the dollar reward.

It should be noted that the assumption of normal distribution was not met for some of the response rates (GS dollar, NGS dollar, and NGS nickel) and RT (GE and NGE reward). Although it has been argued that the normality assumptions can be relaxed for ANOVA except for cases with a small sample size (n<20) [71,72], we employed Box-Cox transformation (λ = -1, inverse transformation) [73] and non-parametric test (Kruskall-Wallis [74]) where appropriate. Overall, the results did not significantly differ from those of the ANOVAs.

### Skin conductance response

**Fig 3:**
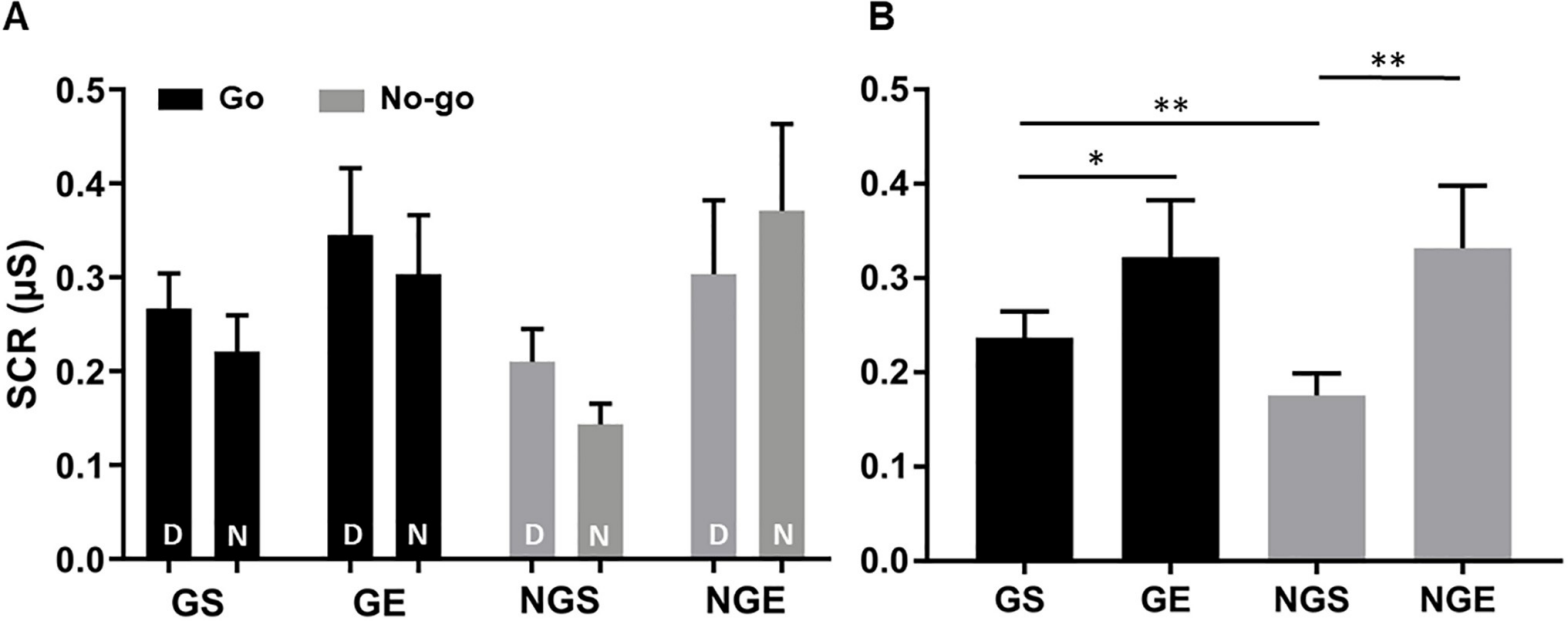
Skin conductance response results. SCR (mean ± s.e.) of dollar (D) and nickel (N) trials (**A**) separated and (**B**) combined. Punishment showed greater SCR than reward whereas SCR was higher for go than no-go trials. GS: go success; GE: go error; NGS: no-go success; NGE: no-go error; D: dollar; N: nickel. * *p* < .05, ** *p* ≤ .01.

We first conducted a 2 × 2 × 2 (Go vs. No-go × Success vs. Error × Dollar vs. Nickel) ANOVA, which showed a significant main effect for outcome (Success vs. Error) (*F*(1,40) = 5.08, *p* = .03) but not for response (Go vs. No-go) or reward value (Dollar vs. Nickel) (*F*’s < 1). None of the interaction effects were significant (*p*’s > .13) (Fig 3A). Post hoc analyses showed the dollar value was associated with greater SCRs than the nickel value in go (*p* = .05, not significant after correction for multiple comparisons) and no-go (*p* = .007) successes, but not in error trials (*p*’s > .3).

We thus combined dollar and nickel trials in a two-way (Go vs. No-go × success vs. error) ANOVA of the SCR (Fig 3B). The results showed a significant effect of outcome (*F*(1,58) = 8.62, *p* = .005) but not of response or outcome × response interaction (*F*’s < 1). In post hoc analyses, GS trials showed significantly higher SCR than NGS trials (*t*(66) = 3.36, *p* = .001). In contrast, GE and NGE trials did not differ in SCR (*p* = .88). GE showed significantly greater SCR than GS (*t*(66) = 2.05, *p* = .04) and NGE showed significantly greater SCR than NGS (*t*(59) = 2.52, *p* = .01). Taken together, error trials showed higher SCR as compared to success trials regardless of action response or economic value. Go showed higher SCR than no-go responses for success trials whereas go and no-go responses did not differ in SCR for error trials.

As the SCR did not follow a normal distribution, we use Box-Cox transformation (λ = .33, cube root transformation) and repeated the ANOVAs, which produced almost identical findings.

### SCR, personality trait, and task performance

**Fig 4:**
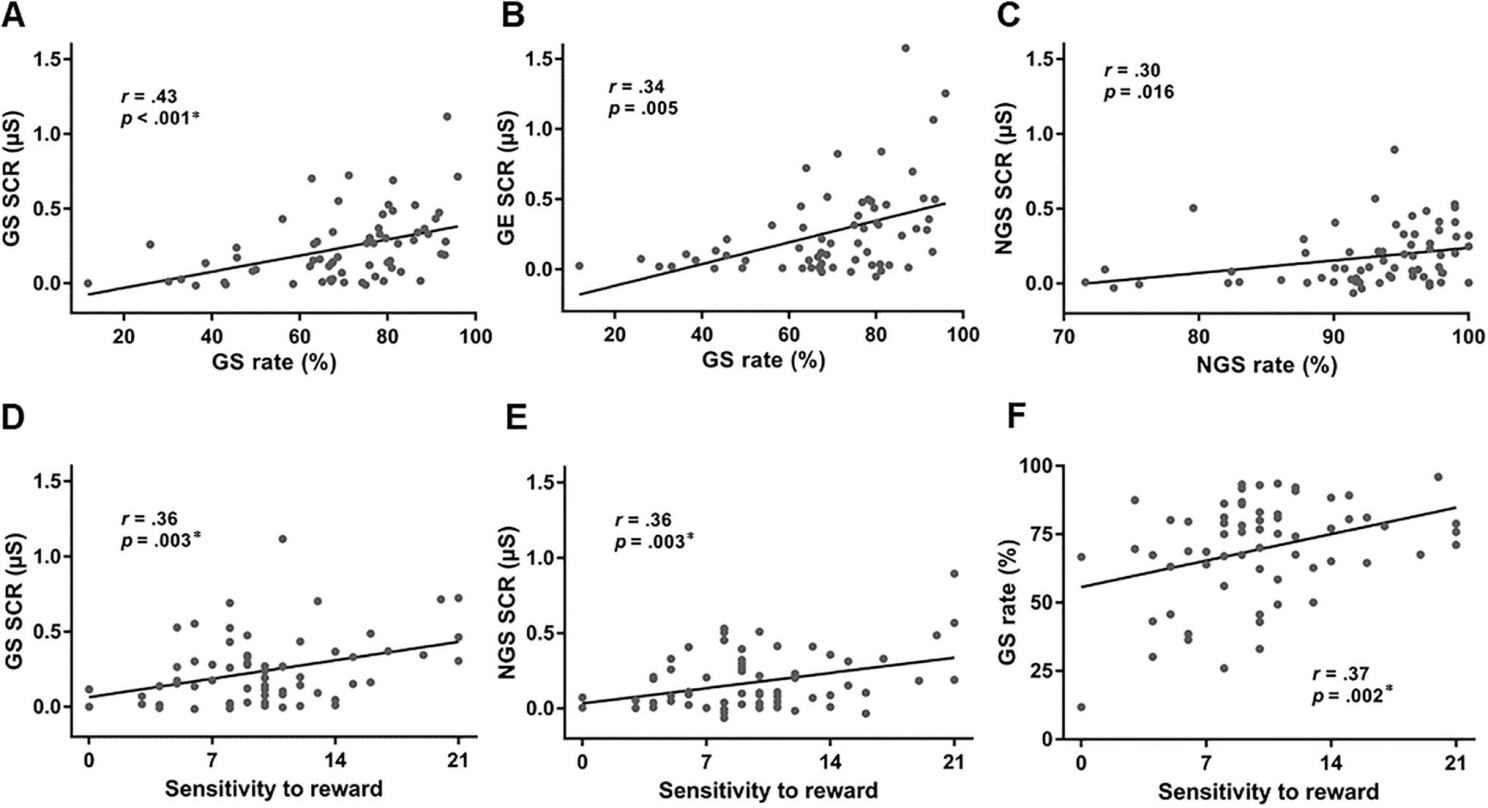
Relationships between SCRs and task performance across subjects. The GS rate was positively correlated with the magnitude of SCR for (**A**) GS, (**B**) GE, and NGS (**C**) trials. Individual sensitivity to reward was positively correlated with the magnitude of SCRs of both (**D**) GS and (**E**) NGS trials as well as (**F**) GS rate. * significant after correction for multiple comparisons.

We examined the relationship between SCR and task performance, including GS and NGS rate and GS and NGE RT. We considered the number of tests–SCR to 4 trial types (GS, NGS, GE and NGE) × 4 outcomes (GS rate, NGS rate, GS RT, and NGE RT)–and evaluated the results with a corrected p of 0.05/(4 × 4) = 0.0031. Across subjects, SCR and GS rate was positively correlated (*r* = .43, *p* < .001) (Fig 4A). At a trend level (using an arbitrary *p*<0.05), SCR of GE trials was correlated with GS rate (*r* = .34, *p* = .005) (Fig 4B) and SCR of NGS trials was correlated with NGS rate (*r* = .30, *p* = .016) (Fig 4C). No other correlations were significant (*p*’s > .06).

We next examined the relationship between SCR and personality traits SR and SP. Again, considering four trial types (GS, NGS, GE and NGE) and two personality measures (SR and SP), we evaluated the results with a corrected p value of 0.05/(4 × 2) = 0.0063. SR was positively correlated with SCRs to both of GS (*r* = .36, *p* = .003) (Fig 4D) and NGS (*r* = .36, *p* = .003) (Fig 4E). No other correlations were significant (*p*’s > .05).

Finally, we examined the relationship between personality traits (SR and SP) and performance (GS rate, NGS rate, GS RT and NGE RT) and evaluated the correlations at a corrected p of 0.05/(4 × 2) = 0.0063. SR was significantly correlated with GS rate (*r* = .37, *p* = .002) (Fig 4F). No other correlations were significant (*p*’s > .02).

### Mediation analysis

Linear regression showed significant correlations among three variables–the SCR of GS trials, GS rate, and SR (Fig 4A, 4D and 4F)–with correction for multiple comparisons. Thus, we focused on these variables in a mediation analysis.

**Fig 5:**
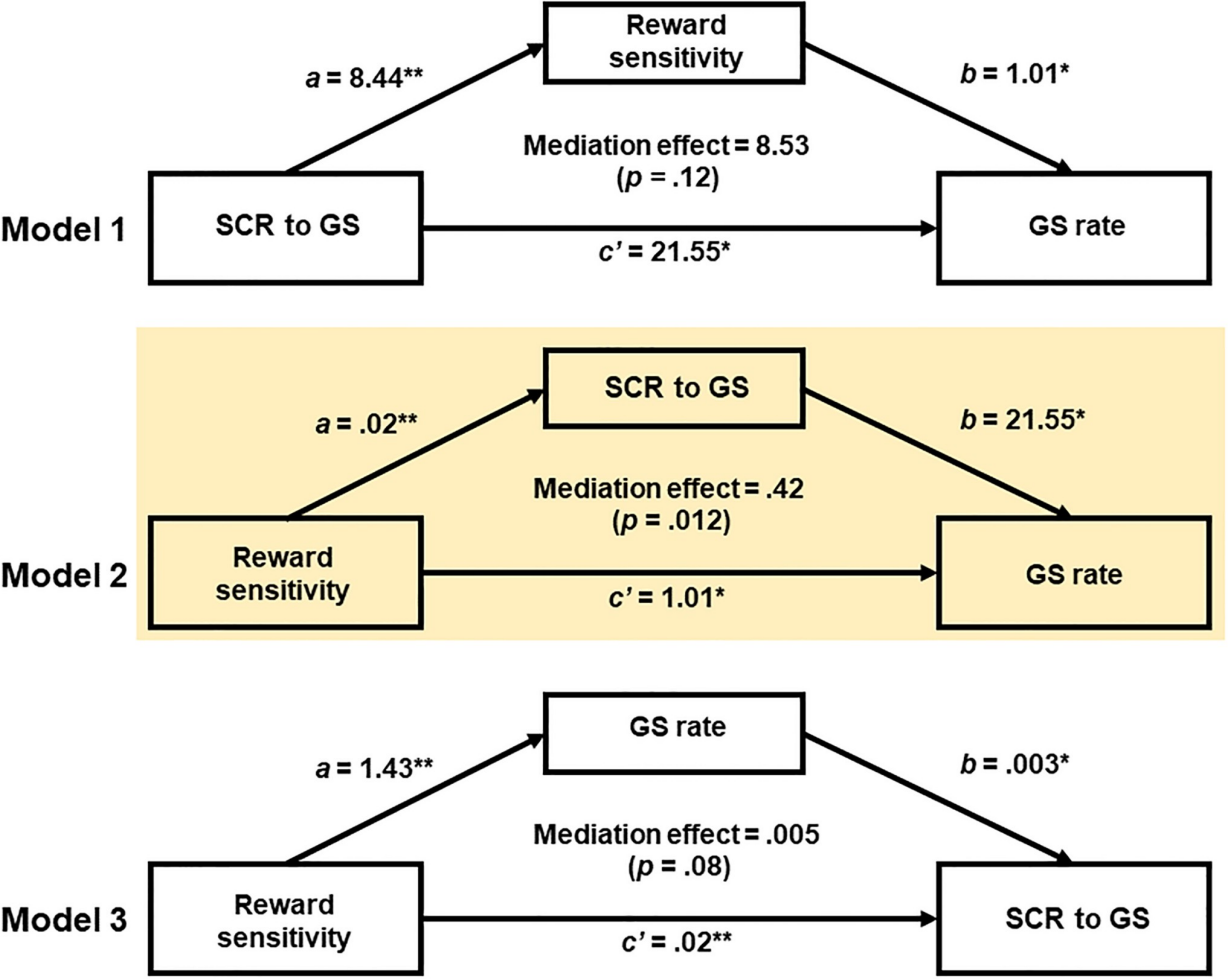
Mediation analysis. (**A**) Model 1 is constructed with skin conductance response (SCR) to GS as the independent variable (X), GS rate as the dependent variable (Y), and sensitivity to reward (SR) as the mediator (M). This model is not significant. (**B**) Model 2 is constructed with X = SR, Y = GS rate, and M = SCR in the GS trials. Model 2 is significant, indicating that SR enhanced task performance through the increase of SCR. (**C**) Model 3 is constructed with X = SR, Y = SCR, and M = GS rate. This model is marginally significant. ** *p* < .01; * *p* < .05.

We tested three specific models. In model 1, SCR contributed to SR, which, in turn, contributed to GS rate: SCR → SR → GS rate. That is, SCR and GS rate served as the independent and dependent variable, respectively, whereas SR served as the mediator. The model was not significant (mediation effect *p* = .12) (Fig 5; Table 1A). In Model 2 (SR → SCR → GS rate) SCR significantly mediated the effect of SR on GS rate (mediation effect (*c*—*c'*) = .42, *p* = .012, 95% confidence interval = [.13 .8]). Specifically, the path coefficient *c* (i.e., X → Y *before* accounting for the mediating effect) was 1.43 (*p* = .002) and the path coefficient *c'* (i.e., X → Y *after* accounting for the mediating effect) was 1.01 (*p* = .03). Thus, the effect of SR on GS rate was weakened but not completely removed after accounting for mediator, indicating that SCR partially mediated SR and GS rate. Model 3 (SR → GS rate → SCR) showed a marginally significant mediation effect (*p* = .08) (Table 1).

**Table 1:** Mediation of SR, SCR to GS, and go success (GS) rate.

	Path *a* (*X* → *M*)	Path b (*m* → *Y*)	Path *c* (*X* → *Y*)	Path *c*’ (*X* → *Y*)	Mediation path (*c*—*c*’)
**A. All subjects**					
Model 1: *X* (SCR) → *Y* (GS rate) mediated by *M* (SR)					
β	8.44	1.01	30.08	21.55	8.53
p-values	0.006	0.033	0.000	0.010	0.123
Model 2: *X* (SR) → *Y* (GS rate) mediated by *M* (SCR)					
β	0.02	21.55	1.43	1.01	0.42
p-values	0.000	0.01	0.002	0.033	**0.012**
Model 3: *X* (GS rate) → *Y* (SR) mediated by *M* (SCR)					
β	1.43	0.003	0.02	0.02	0.005
p-values	0.002	0.036	0.000	0.008	0.084
**B. Female subjects**					
Model 1: *X* (SCR) → *Y* (GS rate) mediated by *M* (SR)					
β	4.34	1.19	30.17	25.00	5.166
p-values	0.39	0.111	0.094	0.162	0.501
Model 2: *X* (SR) → *Y* (GS rate) mediated by *M* (SCR)					
β	0.01	25.00	1.37	1.19	0.177
p-values	0.274	0.162	0.063	0.111	0.465
Model 3: *X* (GS rate) → *Y* (SR) mediated by *M* (SCR)					
β	1.37	0.00	0.01	0.01	0.002
p-values	0.063	0.187	0.274	0.535	0.364
**C. Male subjects**					
Model 1: *X* (SCR) → *Y* (GS rate) mediated by *M* (SR)					
β	10.31	0.78	33.44	25.42	8.018
p-values	0.022	0.165	0.010	0.009	0.270
Model 2: *X* (SR) → *Y* (GS rate) mediated by *M* (SCR)					
β	0.022	25.42	1.34	0.78	0.561
p-values	0.000	0.009	0.042	0.165	**0.014**
Model 3: *X* (GS rate) → *Y* (SR) mediated by *M* (SCR)					
β	1.34	0.01	0.02	0.01	0.008
p-values	0.042	0.112	0.000	0.051	0.110

Mediation analyses showed a significant Model 2 (SR → SCR → GS rate) in all subjects (**A**) and male (**C**) but not female (**B**) subjects.

### Sex differences

For success rate, we conducted an ANOVA with response and reward value as the within-subject factors and sex as a between-subject factor. The result showed significant main effects for sex (*F*(1, 65) = 11.20, *p* < .001), response (*F*(1, 65) = 176.93, *p* < .001), and reward (*F*(1, 65) = 22.75, *p* < .001). There was a significant response × reward interaction (*F*(1, 65) = 39.94, *p* < .001). No other interactions were significant (*p*’s > .27). Post hoc analyses revealed that men showed higher NGS dollar (*t*(65) = 2.05, *p* = .04) and NGS nickel (*t*(65) = 2.85, *p* = .006) rates in men relative to women. For RT, we conducted a similar ANOVA, which showed significant main effects for sex (*F*(1, 43) = 7.05, *p* = .008), response (*F*(1, 43) = 7.86, *p* = .006), and reward (*F*(1, 43) = 20.46, *p* < .001). No interactions were significant (*p*’s > .09). In post hoc analyses, men showed faster RT in both GS dollar (*t*(65) = -2.56, *p* = .01) and GS nickel (*t*(65) = -2.02, *p* = .04) trials compared to women. No other group comparisons were significant (*p*’s > .13). These results suggest that, overall, men performed better than women, showing higher NGS rate despite faster go response.

**Fig 6:**
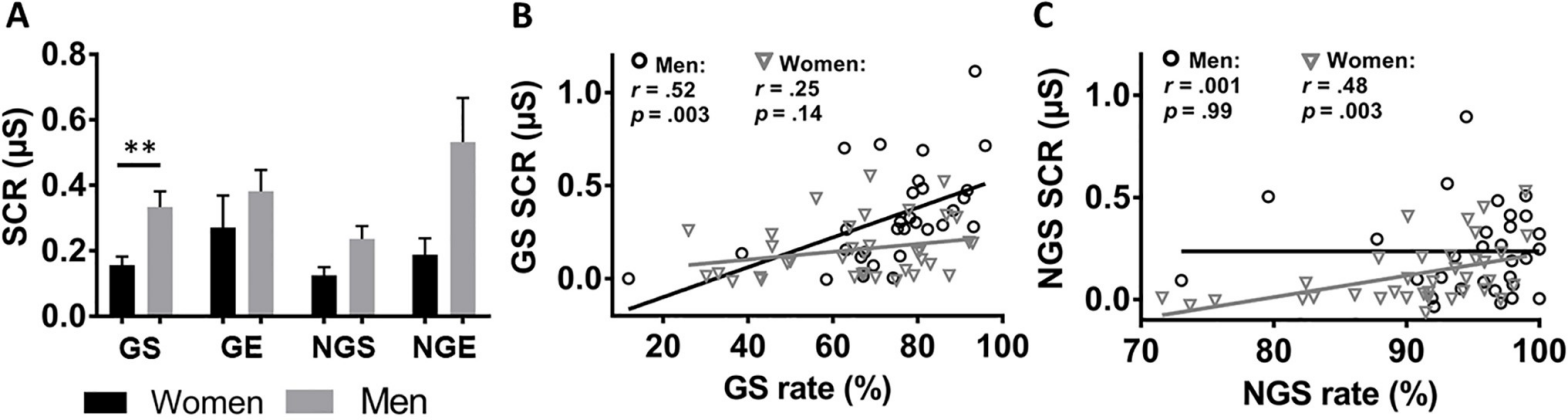
Sex differences in SCRs across trial types. (**A**) Men showed significantly greater SCRs to GS trials with correction for multiple comparisons. (**B**) SCR to GS correlated with GS rate in men but not in women; (**C**) in contrast, SCR to NGS correlated with NGS rate in women but not in men, both significant with correction for multiple comparisons. ** *p* < .01.

We next examined whether the patterns of SCR differed between men and women across different actions and outcomes. In an ANOVA with response and outcome as the within-subject factors and sex as a between-subject factor, there was a significant main effect of sex (*F*(1,57) = 6.89, *p* = .01) but not response × sex (*F*(1,57) = 1.29, *p* = .26), outcome × sex (*F* < 1), or sex × response × outcome interaction effect (*F*(1,57) = 3.6, *p* = .06). In post hoc analyses, men showed significantly greater SCRs than women in all but GE condition (*p* = .37). However, only the sex difference for GS (*p* = .002) survived correction for multiple comparisons (corrected p value of 0.05/4 = 0.012) whereas NGS (*p* = .016) and NGE (*p* = .023) did not (Fig 6A).

Earlier we showed a positive correlation between GS SCR and GS rate across all subjects. This correlation remained significant in men (*r* = .52, *p* = .003) but not in women (*r* = .25, *p* = .14) alone. The slope test confirmed the sex difference (*p* < .05) (Fig 6B). Conversely, the correlation between NGS SCR and NGS rate was significant and stronger in women (*r* = .48, *p* = .003) than in men (*r* = .001, *p* = .99), as also confirmed by a slope test (*p* < .05) (Fig 6C). Next, considering four trial types (GS, GE, NGS and NGE) and two groups of subjects (men and women) and with a corrected p of 0.05/(4 × 2) = 0.0063, we examined the relationship of SCR with reward sensitivity (SR). In men, SR was significantly correlated with SCR to NGS (*r* = .52, *p* = .003), with SCR to GS at a trend level (*r* = .48, *p* = .008) but not with SCR to GE (*r* = .39, *p* = .03) or to NGE (*r* = .42, *p* = .038) trials. None of the same correlations reached significance in women (*p*’s > .3). Thus, physiological arousal was predictive of SR and GS rate in men but not in women; in contrast, physiological arousal was predictive NGS rate in women but not in men.

Finally, we repeated the mediation analyses for women (Table 1B) and men (Table 1C) separately. The results for men followed the pattern of the entire subject group. Model 2 showed a significant mediation effect (*p* = .01). Specifically, SR→SCR path coefficient was significant (*a* = .02, *p* < .001), SCR→GS rate path coefficient was significant (*b* = .25.42, *p* = .009), SR→GS rate path coefficient *before* accounting for the mediator was significant (*c* = 1.34, *p* = .04) but not *after* accounting for the mediator (*c’* = .78, *p* = .17). This indicated a complete mediation of the relationship between SR and task performance by SCR. Model 1 (mediation effect = 8.02, *p* = .27) and 3 (mediation effect = .008, *p* = .12) were not significant. No model showed a significant mediation effect in women (Model 1: mediation effect *c*—*c'* = 4.47, *p* = .55; Model 2: *c*—*c'* = .12, *p* = .58; Model 3: *c*—*c'* = .002, *p* = .49).

## Discussion

Errors as compared to successes were associated with elevated SCRs during both go and no-go responses. SCRs were higher for go (GS) relative to no-go success (NGS) but indistinguishable for errors. Dollar reward elicited greater SCR than nickel reward in the successful trials, indicating a potential reward magnitude effect. SCR was also positively correlated with GS and NGS rate as well as trait sensitivity to reward (SR). Mediation analyses showed that SCR served as a mediator of the relationship between SR and GS rate. Thus, SR facilitated physiological arousal to potentially rewarding actions, which, in turn contributed to enhanced behavioral performance. In sex differences, men exhibited greater SCR, which was more predictive of go success rate, relative to women. In contrast, SCR was more predictive of no-go success rate in women. Together, the findings highlighted the influence of behavioral and motivational factors including reward outcome and action type as well as individual differences in sex and reward sensitivity on physiological arousal during GNG task performance.

### SCR to error/punishment

SCRs were enhanced to monetary loss as compared to gain regardless of the underlying action or reward value, consistent with studies of healthy individuals showing higher SCRs to punishment relative to reward in the IGT [40,75], ultimatum game [22], decision making [20], and reward GNG [76] tasks. The anticipation of punishment also elicited greater SCRs to the selection of disadvantageous relative to advantageous decks during the IGT [43,77,78]. Thus, as an autonomic indicator of current or anticipated loss, SCR likely plays a role in the physiological mechanism that responds to negative stimuli or outcomes to guide future behaviors [1,23,79]. Indeed, punishment for errors has been associated with both increases in SCR and post-error slowing in a two-choice reaction time task [25] as well as fewer no-go errors in a GNG task [76]. The negativity bias may provide a mechanism whereby reward losses enhance arousal and facilitate the allocation of attentional resources to motivationally relevant events [23]. In this attentional model, physiological responses are posited to be modulated by the absolute losses rather than loss sensitivity. Another possibility is that interoceptive signals may generate anticipatory SCRs prior to risky decisions, as demonstrated with the IGT [41], although the risk primarily involves response speed in the GNG task compared to reasoning and strategizing in the IGT.

In clinical populations, failures to elevate SCR to punishment have been implicated in deficient inhibitory control and risky behaviors. For instance, those who exhibited psychopathic traits [44,80,81] or engaged in substance abuse [82–84] showed lower SCR to punishment than healthy individuals. Compared to control subjects, children with attention deficit hyperactivity disorder demonstrated reduced SCR to no-go errors in a sustained attention task and this reduction was associated with poorer inhibitory performance [85]. Together, SCR reflects physiological arousal in response to aversive events and to the anticipation of negative reinforcers, and alterations of these physiological responses may be associated with cognitive, affective, and behavioral deficits in clinical populations.

### SCR and approach vs. avoidance

Go successes showed higher SCR than no-go successes, suggesting enhanced physiological arousal in incentivized approach vs. avoidance behavior. This finding is supported by previous reports of greater SCR to go than no-go responses in the GNG tasks in which go and no-go trials were, unlike the current study, equiprobable and not incentivized [26,27]. Higher SCR to go than to no-go trials may reflect the activities of noradrenergic neurons to support the initiation and execution of motor actions [86]. As shown in work using an incentive force task, SCR increased linearly with both reward value and the amount of physical force participants exerted during motor responses to achieve monetary gains [87]. An imaging study found skin conductance activity to be synchronous in onset with brain activations and exhibiting a transient pattern following repetition of movements, suggesting the dependence of physiological arousal on task difficulty [88]. Imagined movements such as swimming also elicited an increase in SCR, particularly at the beginning of imagination [89]. Recordings from the locus ceruleus, the main noradrenergic nucleus that supports arousal and regulates sleep-wake cycle, showed increased neuronal activities during motivated movements but not non-movement [90], in agreement with higher SCR during go than no-go trials in the current study. While these reports corroborate our results, it is plausible (though unlikely) that lower SCR in the no-go trials may have reflected the lack of response due to inattention rather than cue instruction. Taken together, SCR may be associated with the execution of a movement and the amount of effort associated with such movement. The current findings highlight the differential patterns of SCR to rewarded action vs. inhibition of action.

### SCR, behavioral performance, and personality trait

As skin conductance activity is thought to index attention [91,92], greater SCRs in association with higher GS and NGS rates suggested the benefits of attention and arousal in enhancing behavioral performance. Orienting attention to relevant stimuli facilitates perceptual and motor processing [91,93,94], goal-directed motor control [95], interference resolution in Stroop [96], as well as response inhibition in the flanker [97] and GNG [98,99] tasks. SCRs to auditory attention predicted reaction time slowing in a concurrent visual response task, indicating that enhanced SCR and attention to a primary target depleted cognitive resources that could be allocated elsewhere [100]. Task difficulty appears to affect physiological arousal, as SCRs increase with higher cognitive load during the N-back task [101,102], sentence repetition task [103], and dual-task interference [104]. Further, shifting attention toward reinforcing stimuli may enhance arousal [105] as well as activate preparatory motor mechanisms [106], in support of a link between attention, enhanced arousal and behavioral performance. It should be noted that as the literature is sparse on the relationship between SCR and behavioral performance in the GNG task, the current findings need to be interpreted with caution. Many other factors are likely at play and more work is needed to establish a conceptual framework regarding how individual differences in physiological arousal may predict behavioral performance.

Carver and White (1994) [107] proposed that the appetitive motivational system is activated by the anticipation of incentives. The intensity of this activation, reflected in arousal and partly determined by trait sensitivity, promotes reward-seeking behavior [4,108]. These relationships indicate interdependence among physiological, dispositional, and behavioral factors. Previous studies have used mediation analyses to show that regional brain activation and impulsivity mediated the relationship between reward sensitivity and behavior during incentivized working memory [109] and risky driving behavior [110]. Here, we showed that higher sensitivity to reward (SR) was associated with both greater SCR and response accuracy. Our mediation model further suggested that SR facilitated go response accuracy at least partly through the enhancement of SCR. This finding is in keeping with the proposal that sympathetic arousal contributes to goal-directed effort [111] and that changes in SCR to salient events may accompany activations of the motivation circuits, including the insula and dorsal anterior cingulate cortex [111–113], to facilitate goal-directed behaviors [3,111].

Other studies have reported a negative impact of heightened SR on task performance requiring inhibitory control, seemingly inconsistent with the current finding on the no-go trials. For instance, SR was positively correlated with the stop-signal reaction time, suggesting deficient inhibition, in a stop signal task [114] and with more frequent risky decisions in the IGT [50,115] and Columbia Card task [116]. It is likely that differences in behavioral contingencies may account for these discrepancies. We rewarded both go and no-go success whereas Avila and Parcet (2001) [114] did not incentivize task performance. Further, the reward contingencies are different between the gambling paradigms and the current GNG task and may influence the effects of SR on performance. Finally, while the current findings pointed to a psychophysiological link between the appetitive motivational system and rewarded actions, it should be noted that a mediating effect does not imply a causal relationship, and the issue whether physiological arousal or interoception plays a causal role in behavior remains to be investigated [1,117].

It is worth pointing out that our hypothesis that heightened SP would be associated with elevated SCRs as well as correct response rate of the no-go trials was not confirmed. As there were twice as many go as no-go trials, participants may be biased toward go action, resulting in a weakened relationship between SP and behavioral as well as skin conductance measures of no-go response. Further, SP was not significantly correlated with SCRs to either go or no-go errors, consistent with previous work showing that physiological arousal did not reflect loss sensitivity in a rewarded decision-making task [20].

### Sex differences

Men exhibited higher SCRs than women across trial types, at odds with the common conception that women are more easily aroused by emotional and salient events, as compared to men [55]. Studies showed higher SCR in women relative to men when expecting electric shocks [118], preparing for public speaking [19], and viewing emotionally negative images [49], suggesting a higher harm avoidance tendency than men [60]. This sex difference appeared to be especially pronounced in stressful situations [119] and in individuals with depression and anxiety disorders [120]. However, others have reported the opposite patterns of response during exposure to salient visual and olfactory stimuli [49,53,54], as well as no sex differences during exposure to either negative or positive images [13,57,58,121,122].

Studies have highlighted greater female sensitivity during passive exposure to negative stimuli and reported higher reward-seeking responses in men during goal-directed behaviors. In a meta-analysis examining trait sensitivity and measures of approach and avoidance behaviors, men exhibit greater motivation to pursue reward than women whereas women exhibit greater motivation to avoid punishment than men [60]. In studies involving financial incentives, men were more motivated by monetary gains and less willing to share reward or accept small offers relative to women in laboratory paradigms [123,124]. Further, in brain imaging men as compared to women showed significantly greater activation to emotionally positive stimuli, including financial incentives, in the amygdala and visual cortices [58,125,126]. Thus, the current findings of enhanced SCR and behavioral performance may have reflected a stronger drive to seek reward in men than in women.

Compared to women, men showed higher SCR to both GS and NGS trials. Interestingly, while men exhibited a more robust relationship between SCR and SR as well as GS rate, women demonstrated a stronger relationship between SCR to NGS trials and NGS rate. The findings suggest the importance in examining sex-specific characteristics in physiological arousal in conjunction with approach and avoidance behavior. In particular, despite a lack of sex difference in SR, there was a closer relationship between SR and physiological arousal as well as reward-driven behavior in men than women. As these sex differences may be specific to behavioral contingencies, more work is needed to explore how task variables, including go/no-go ratio and a predominantly punishing scheme, may influence arousal.

## Conclusions

Combining a reward GNG task, recording of electrodermal responses, and measures of trait sensitivity, we showed that physiological arousal depended on task outcome, the underlying action, and individual differences including reward sensitivity and sex. Skin conductance response to monetary reward partially mediated the relationship between reward sensitivity and go success rate. These findings suggest interdependent links between the autonomic nervous activity, personality, and goal-directed behavior and may help us better understand the biological bases of approach and avoidance behaviors in health and illness.
